# Supplementary material for: A flexible artificial chemosensory neuronal synapse based on chemoreceptive ionogel-gated electrochemical transistor
Source: Nat Commun. 2023 Feb 14;14:821. doi: 10.1038/s41467-023-36480-6 (PMC9929093; doi:10.1038/s41467-023-36480-6)
Supplement: Supplementary file 1 — Supplementary Information [file 41467_2023_36480_MOESM1_ESM.pdf]

# Supplementary information

# A flexible artificial chemosensory neuronal synapse based on chemoreceptive ionogel-gated electrochemical transistor

*Hamna Haq Chouhdry*[1,2], *Dong Hyun Lee*[3], *Atanu Bag*[3,4✉] *and Nae-Eung Lee*[1,2,3,4,5,6,7✉]

[1] SKKU Advanced Institute of Nano Technology (SAINT), Sungkyunkwan University, Suwon, Gyeonggi-do 16419, Republic of Korea

[2] Department of Nano Science and Technology, Sungkyunkwan University, Suwon, Gyeonggi-do 16419, Republic of Korea

[3] School of Advanced Materials Science & Engineering, Sungkyunkwan University, Suwon, Gyeonggi-do 16419, Republic of Korea

[4] Research Centre for Advanced Materials Technology, Sungkyunkwan University, Suwon, Gyeonggi-do 16419, Republic of Korea

[5] Samsung Advanced Institute for Health Sciences & Technology (SAIHST), Sungkyunkwan University, Suwon, Gyeonggi-do 16419, Republic of Korea

[6] Institute of Quantum Biophysics (IQB), Sungkyunkwan University, Suwon, Gyeonggi-do 16419, Republic of Korea

[7] Biomedical Institute for Convergence at SKKU (BICS), Sungkyunkwan University, Suwon, Gyeonggi-do 16419, Republic of Korea

✉email: abag@skku.edu; nelee@skku.edu

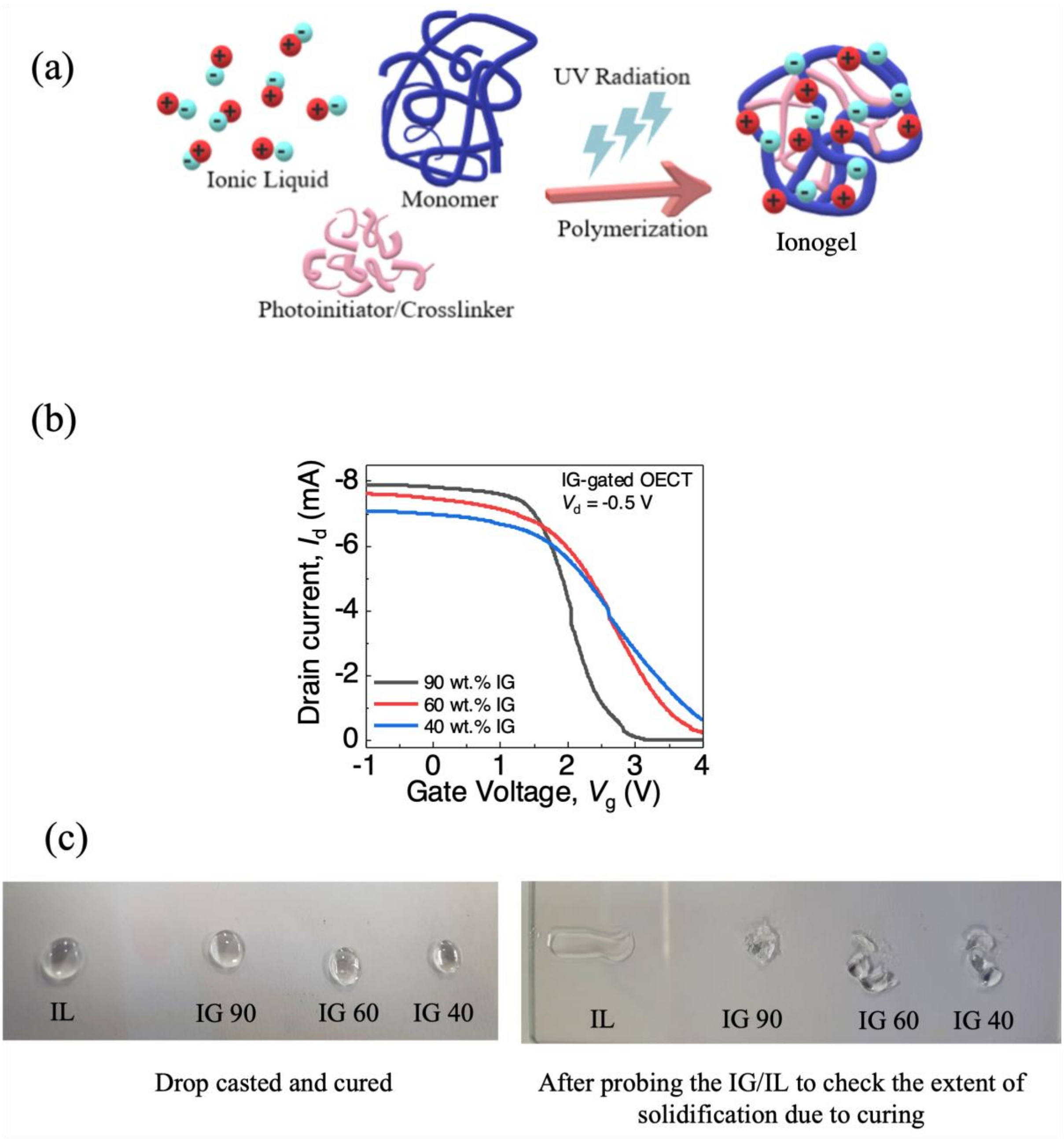


**Fig. S1.** (a) Schematic diagram of IG preparation. (b) Transfer characteristics of the IG-gated devices with different IL concentrations in the IG (90 wt.%. 60 wt.%, 40 wt.% IL). (c) Comparison of different IG compositions according to gel formation properties. The IG (90 wt.% IL) forms a soft gel-like structure, while the IGs (60 and 40 wt.% IL) have a more solidified form as the relative amount of monomer is greater, resulting in a highly cross-liked structure.

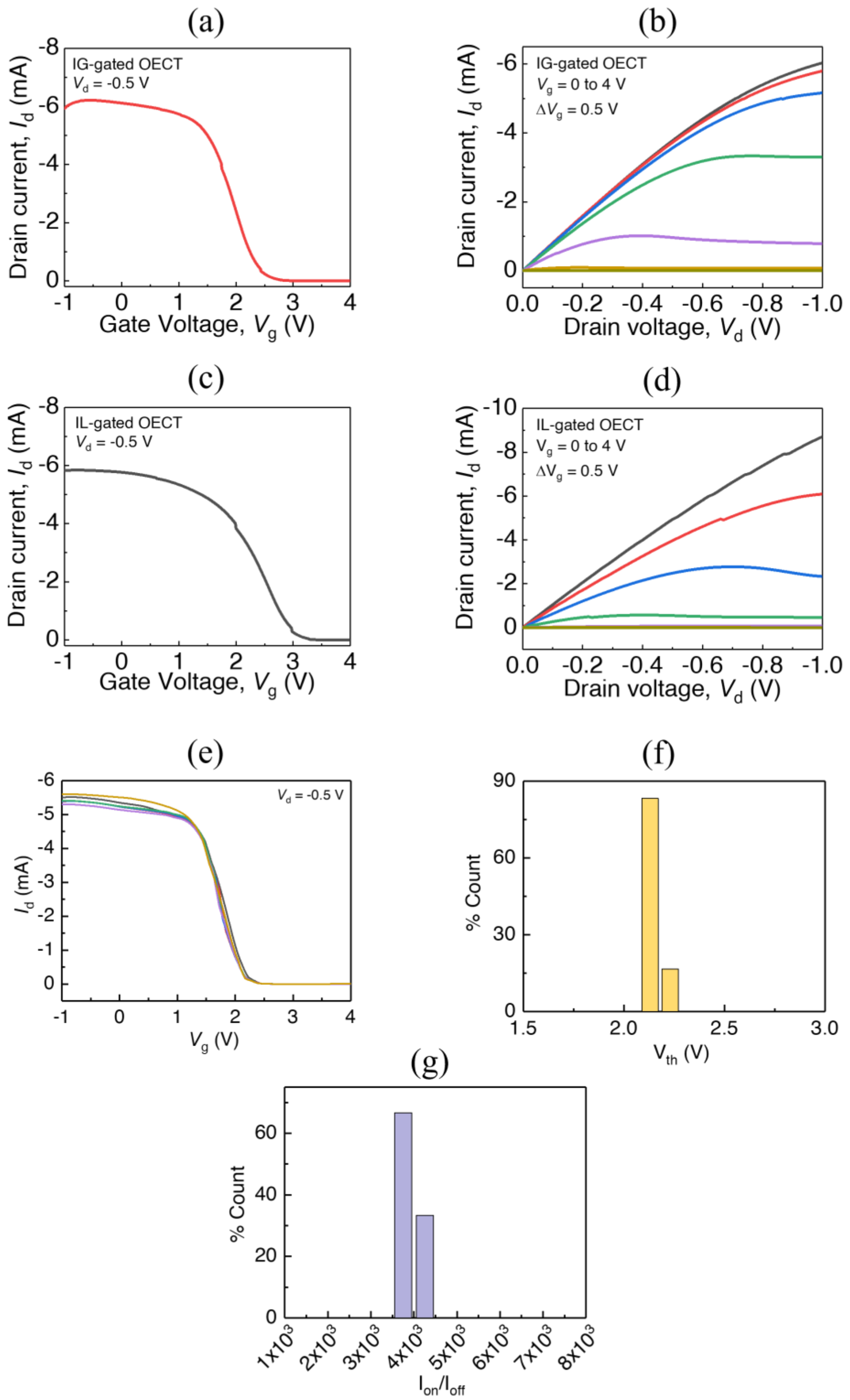


**Fig. S2.** (a, c) Transfer and (b, d) output characteristics for IG (90 wt.% IL)- and IL-gated devices. (e) Transfer characteristics of six IG (90 wt.% IL)-gated devices fabricated on a same substrate. (f) threshold voltage ($V_{th}$) and (g) current on/off ratio ($I_{on}/I_{off}$) of the six devices shown in (e).

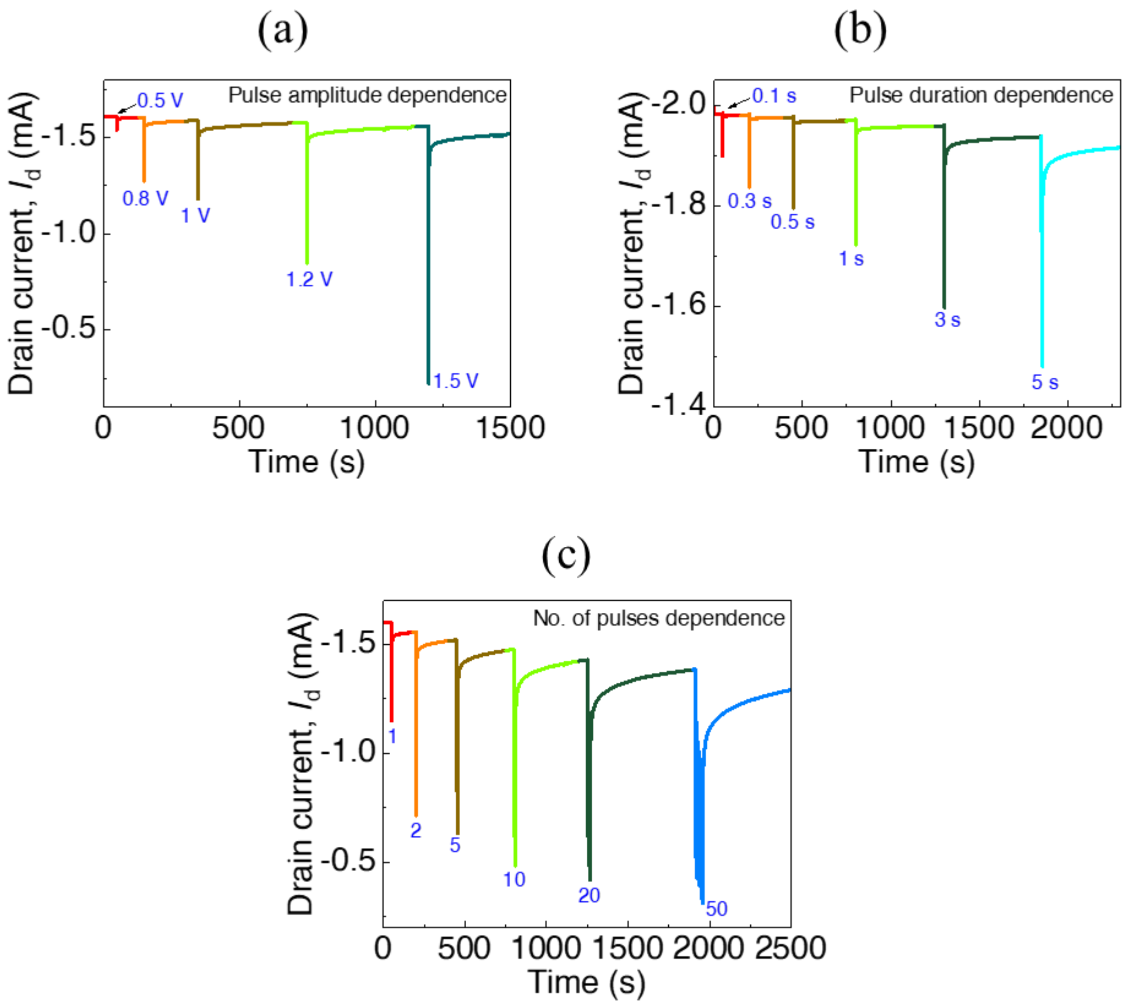


**Fig. S3**. Dependence of PSC (drain current, $I_d$) on electrical pulsing (i.e., electrical stimuli) with varying (a) amplitude, (b) duration, and (c) number of pulses for the IL (90 wt.% IL)-gated device.

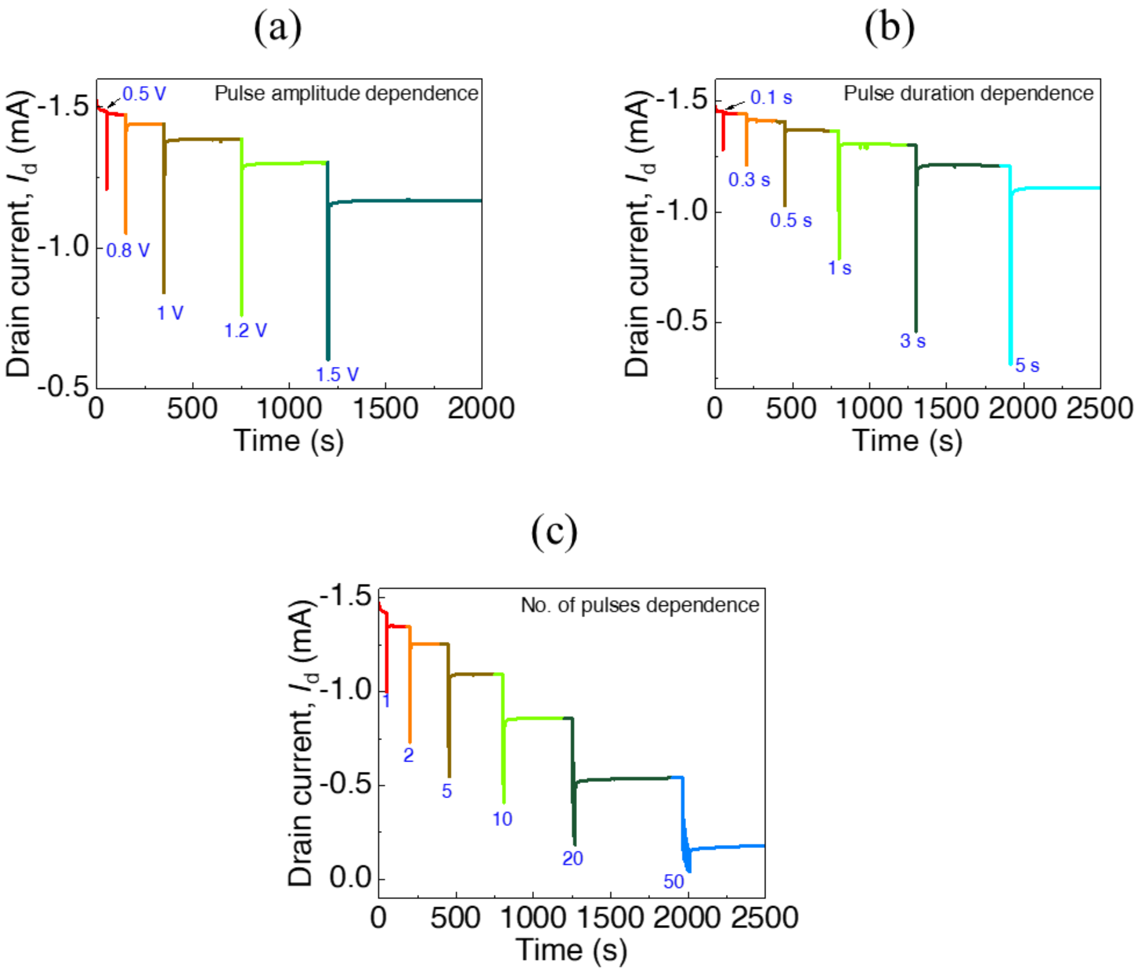


**Fig. S4.** Dependence of PSC (drain current, $I_d$) on electrical pulsing (i.e., electrical stimuli) with varying (a) amplitude, (b) duration, and (c) number of pulses for the IG (90 wt.% IL)-gated device.

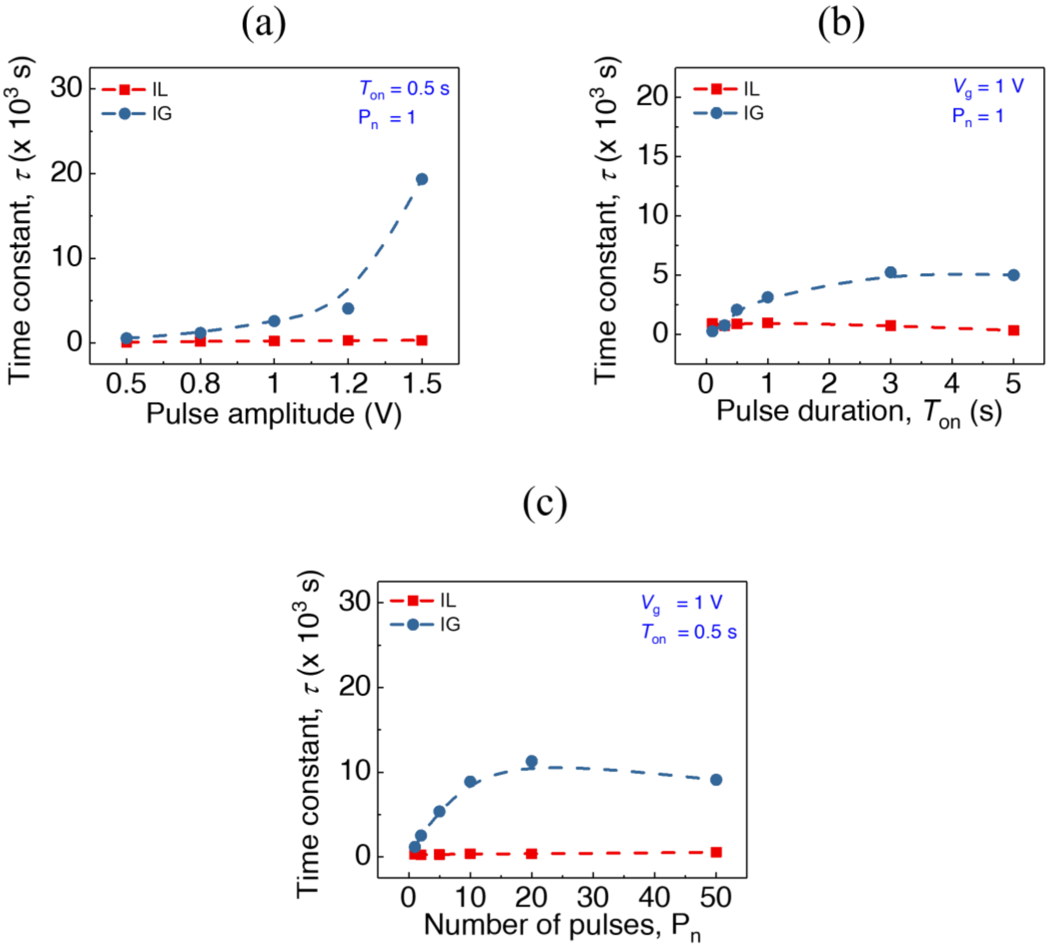


**Fig. S5.** Analyzed decay time constant (τ) for both IL and IG (90 wt.% IL)-gated devices (a) at different amplitudes (b) durations and (c) number of pulses of electrical stimuli (estimated for data in **Fig. S3** and **S4**). The theoretical decay time constant was calculated employing the following equation for exponential decay function:

$$PSC(t) = PSC_{in} + (PSC_0 - PSC_{in})\exp\left(\frac{-t}{\tau}\right)$$

Where *t* is the time after the electrical stimulation, PSC(*t*) is PSC at time *t*, $PSC_{in}$ is the PSC before electrical stimulation, and $PSC_0$ is the PSC at time = 0. $\tau$ is the decay time constant which can give us information on the retention times. Large decay constants refer to longer retention times and slow decay of the current to its initial state.

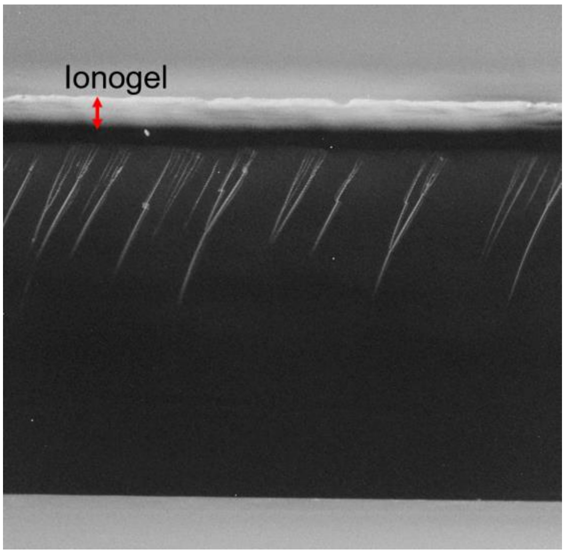


**Fig. S6.** Optical microscope image of IG (90 wt.% IL) layer (thickness of approximately 152 μm) deposited on glass substrate.

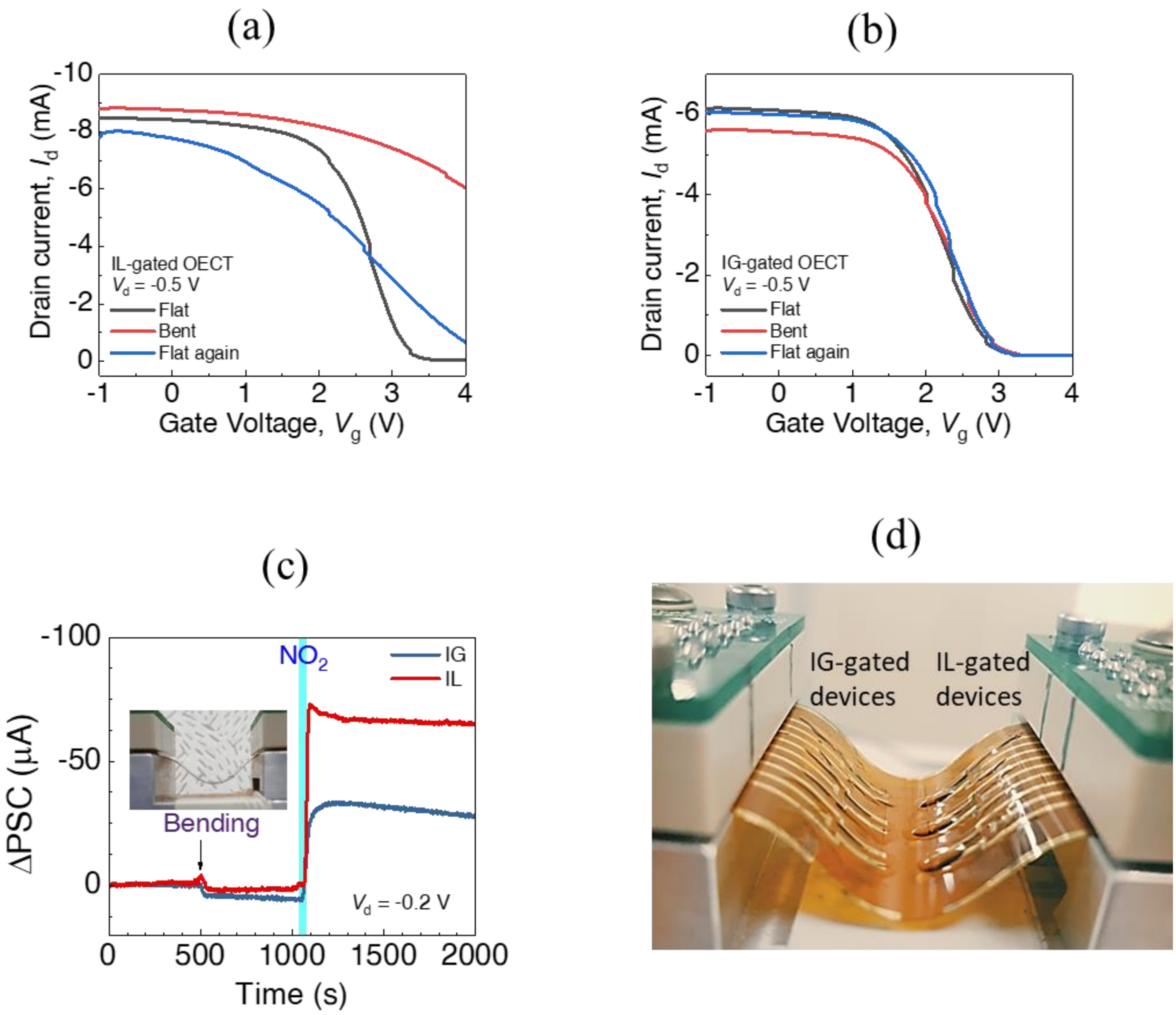


**Fig. S7. Electrical characterization of the IG- and IL-gated OECTs under bending.** The transfer curves ($I_d$ vs. $V_g$) demonstrate instability in the bent IL-gated device (a) but a stable behavior in the IG (90 wt.% IL)-gated device (b) upon bending. (c) The time-dependent ΔPSC data from the bent IL-gated device show a larger ΔPSC change compared with that of IG-gated device upon bending. (d) The IL on the bent IL-gated devices (three devices on the left side of the array) exhibits flowing characteristics over the substrate compared with IG-gated devices (three devices on the right side of the array) due to its liquid nature under bending.

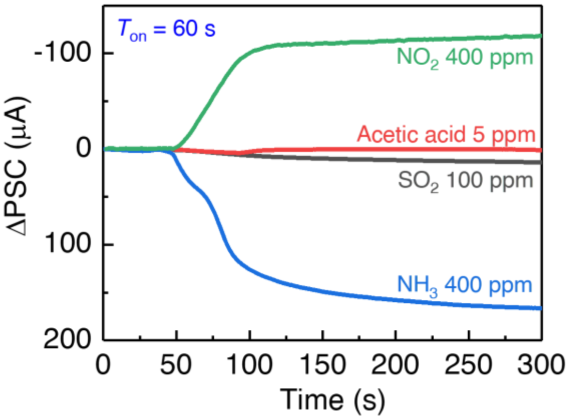


**Fig. S8.** Gas sensing property of the IG (90 wt.% IL)-gated OECT towards four different analytes of $NO_2$, $NH_3$, $SO_2$, and acetic acid. The device showed distinct responses towards $NO_2$ and $NH_3$, while we observed relatively small responses towards $SO_2$ and acetic acid.

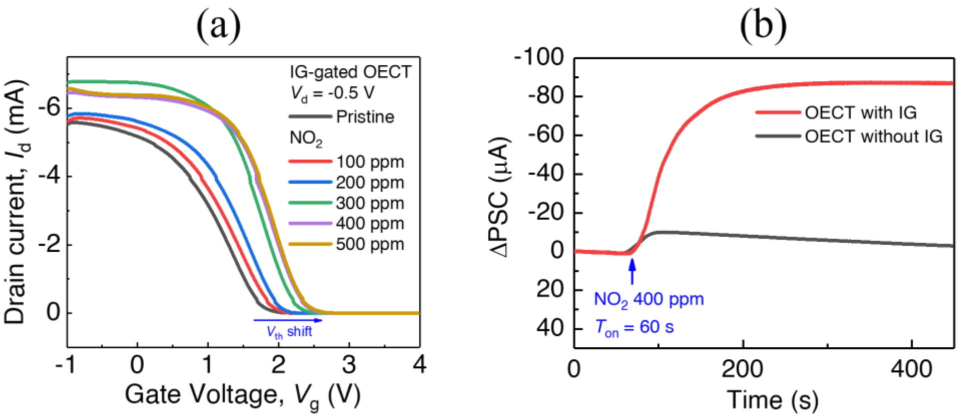


**Fig. S9.** (a) Transfer characteristics of IG (90 wt.% IL)-gated OECT after exposure to increasing concentrations of $NO_2$ (100–500 ppm) with an exposure time of 30 s. (b) Comparison of the responses of the IG (90 wt.% IL)-gated OECT and an OECT without any IG.

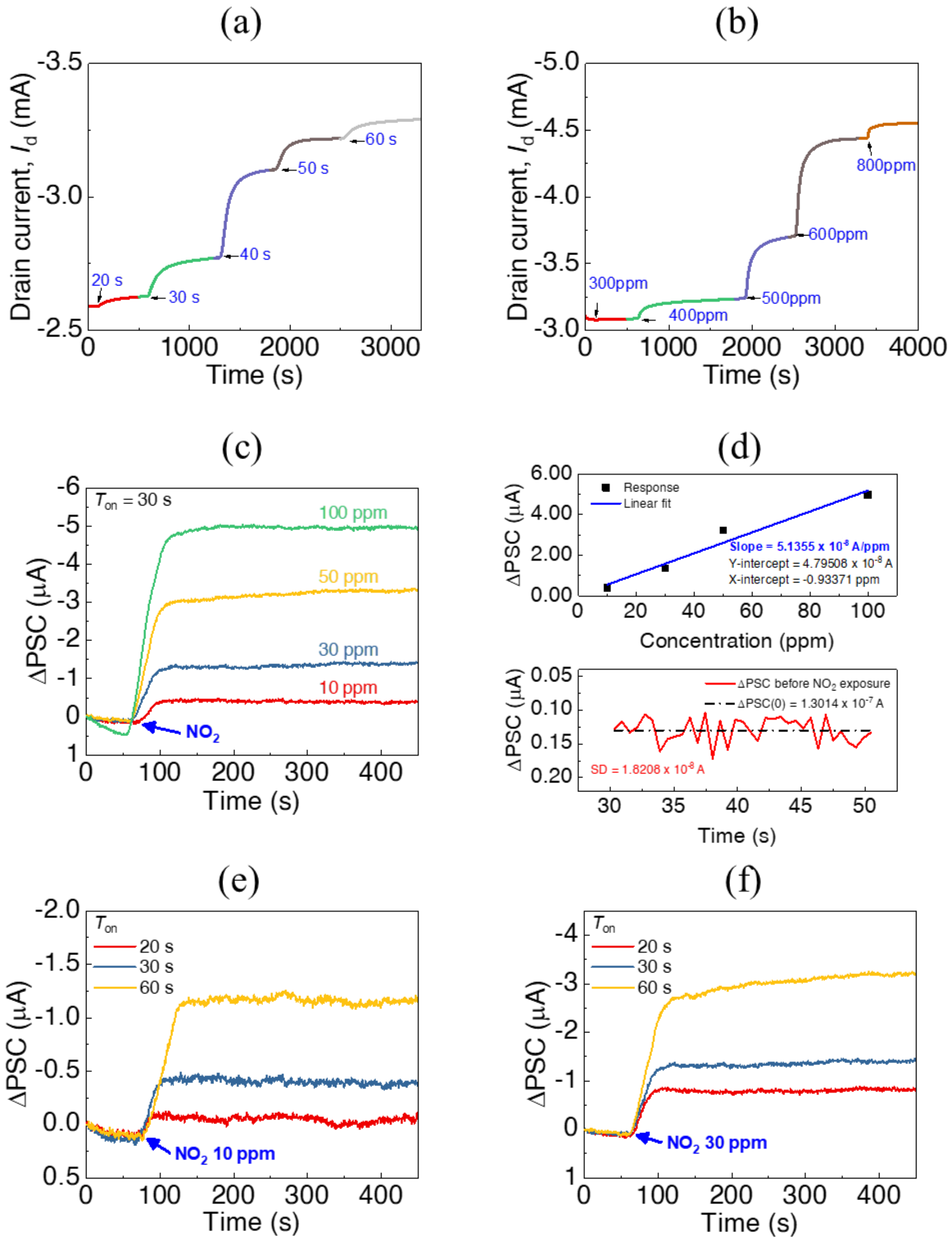


**Fig. S10.** (a) Time-dependent measurement of the drain current ($I_d$) upon exposure to increasing durations of chemical pulses at a $NO_2$ pulsing concentration of 400 ppm. (b) Time-dependent measurement of $I_d$ upon exposure to increasing $NO_2$ concentrations in a step-like manner from 300 to 800 ppm, with a gas pulse duration of 30 s. (c) Time-dependent response (ΔPSC) of the ACNS towards the low concentration of $NO_2$ (10, 30, 50 and 100 ppm) with a pulse duration of 30 s. (d) The *upper* panel shows the linear fitting of response versus concentration plot. The

*bottom* panel shows the average value of ΔPSC before $NO_2$ exposure (i.e., ΔPSC(0)). Calculation of limit of detection (LOD) from the data showing the fitted responses of the device. The theoretical LOD was calculated as: $LOD\ (ppm) = \frac{\Delta PSC(0)+3\times SD-y\text{-}intercept}{slope}$ = 2.66. Dependence of the device response (ΔPSC) on the pulse duration (20, 30 and 60 s) of (e) 10 ppm and (f) 30 ppm $NO_2$ pulses.

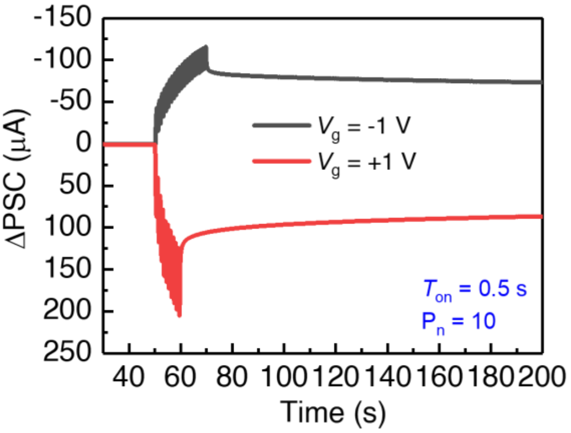


**Fig. S11.** Comparison of the device responses (ΔPSC) under positive and negative electrical gate pulsing showing opposite behaviors. The positive gate bias pulsing implies the penetration of cations while the negative gate bias pulsing implies the penetration of anions into the channel.

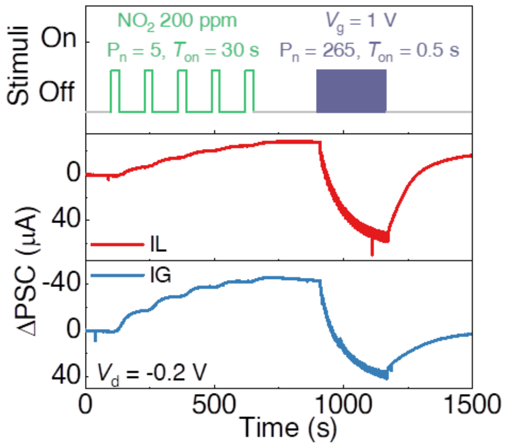


**Fig. S12.** Comparison of change in the ΔPSC values of IG (90 wt.% IL)- and IL-gated devices with chemical pulsing, followed by electrical pulsing, showing longer retention in the IG-gated device compared with the IL-gated device after electrical pulsing.

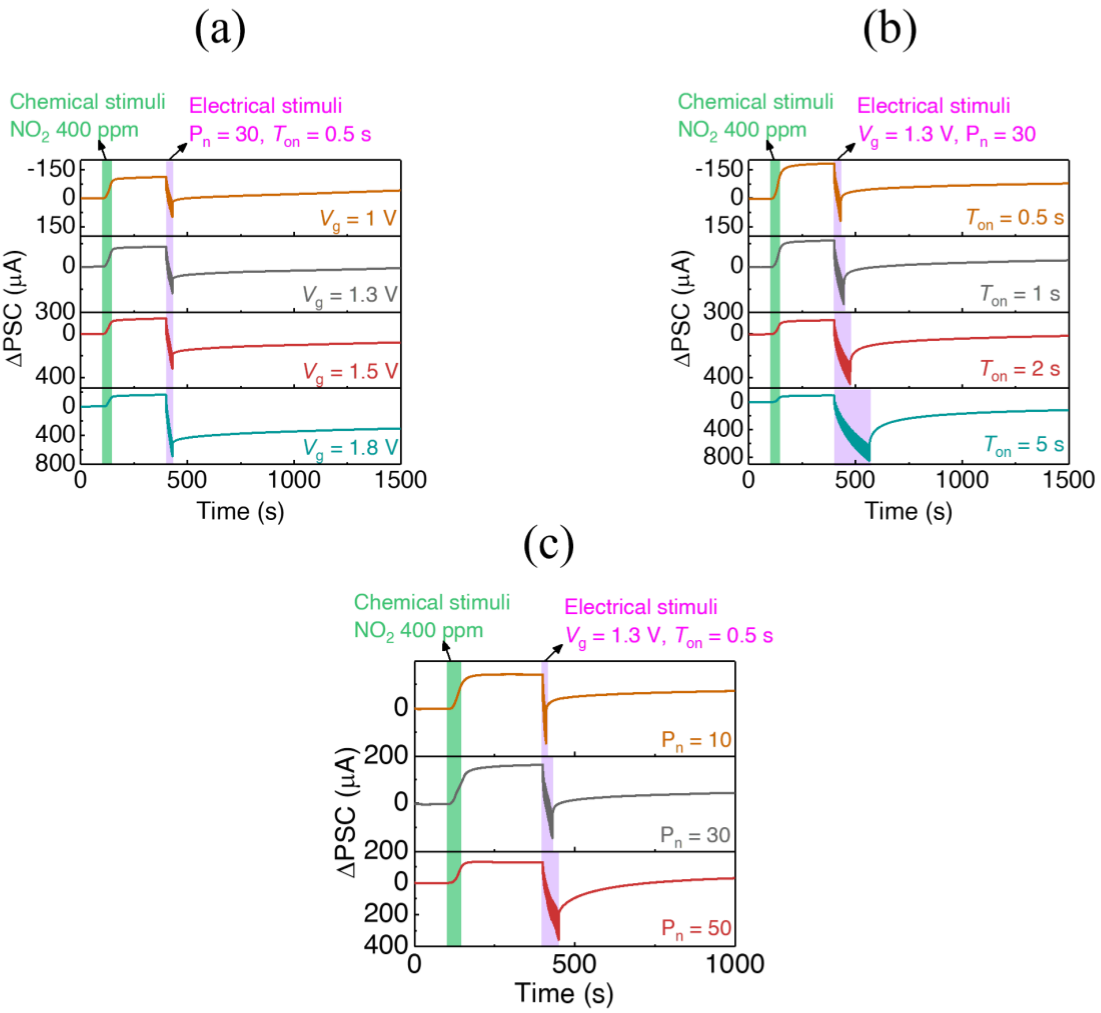


**Fig. S13.** Time-dependent measurement of ΔPSC from the IG (90 wt.% IL)-gated device, with excitatory chemical stimuli ($NO_2$ 400 ppm, $T_{on}$ = 40 s and $P_n$ = 1) followed by inhibitory electrical stimuli with different (a) pulse amplitude ($V_g$), (b) pulse duration ($T_{on}$), and (c) pulse number ($P_n$ = 10, 30, and 50).

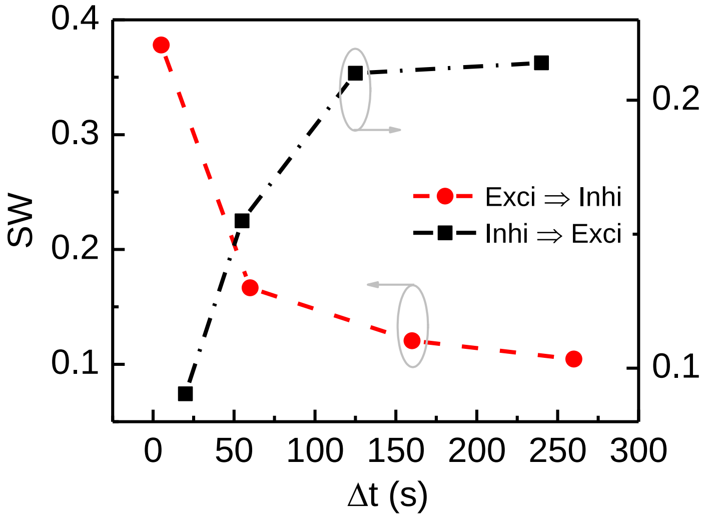


**Fig. S14.** Observed SW values in Fig. 4f without normalization.

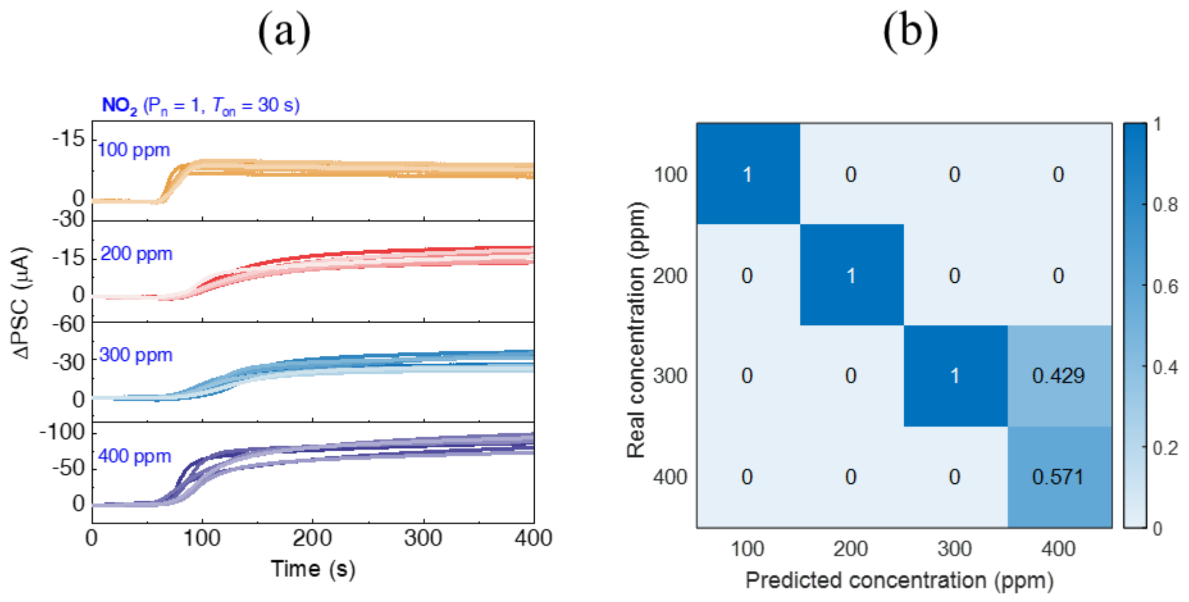


**Fig. S15.** (a) Time-dependent response (ΔPSC) of IG (90 wt.% IL)-gated OECT under chemical stimulation ($NO_2$ with $T_{on}$ = 30 s) with increasing the concentration from 100 to 400 ppm. Repeated measurements were performed to get eight concentration-dependent responses for each case. (b) Normalized confusion matrix showing the classification performance (test accuracy 81.2%) of a single device to 4 different concentrations of $NO_2$ gas (100, 200, 300, and 400 ppm) after training with a ML model (SVM) in Classification Learner. The colored scale bar shows the test accuracy of the classification.

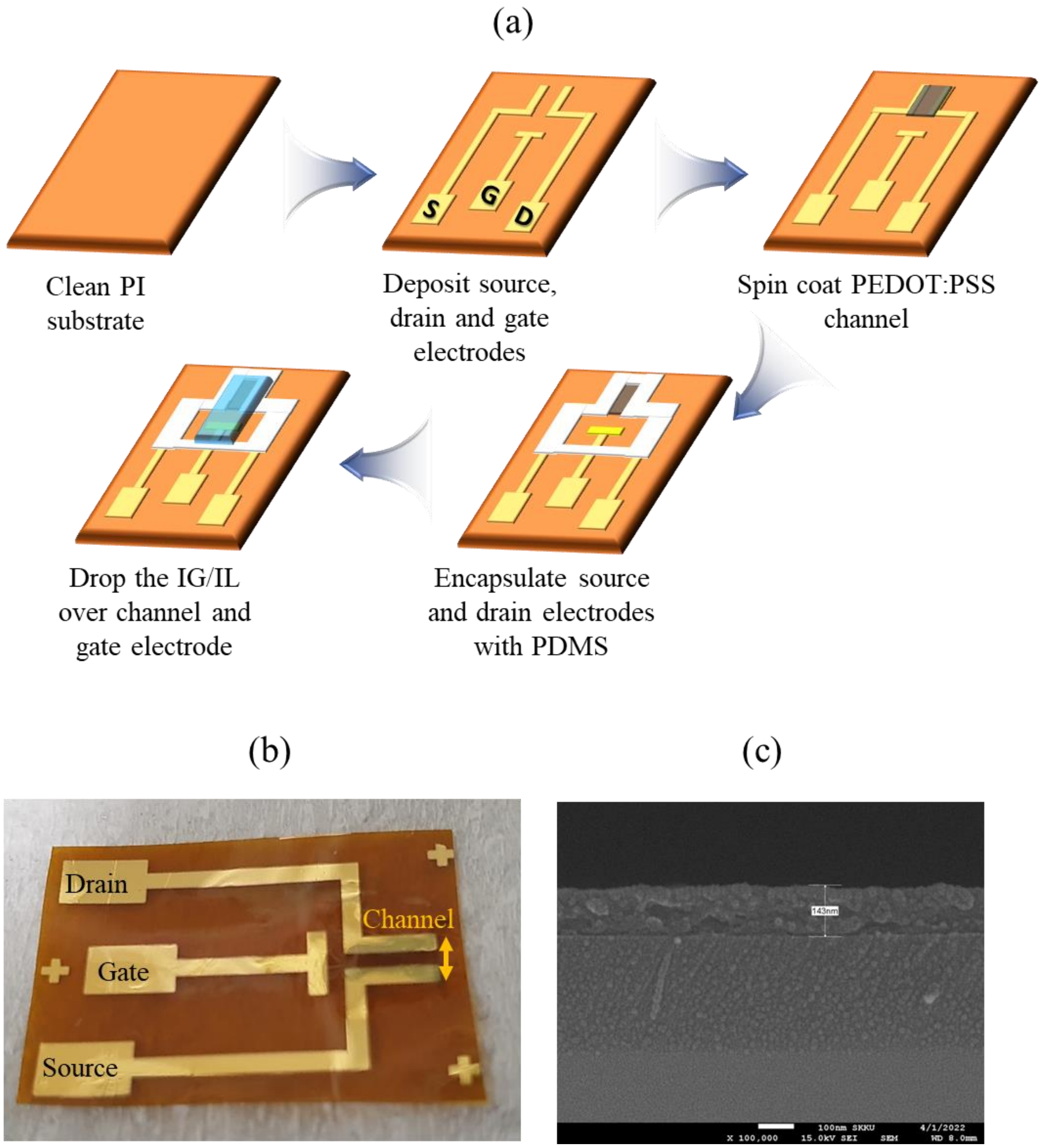


**Fig. S16.** (a) Schematic of the fabrication steps. (b) Photograph of the fabricated device and (c) cross-sectional FE-SEM image of PEDOT:PSS channel layer (thickness of approximately 143 nm).
